# Healthcare consumption of patients with left ventricular assist device: real-world data

**DOI:** 10.1007/s12471-024-01885-5

**Published:** 2024-08-14

**Authors:** Lena Bosch, Peter-Paul M. Zwetsloot, Maaike Brons, Gerardus P. J. van Hout, Manon G. van der Meer, Mariusz K. Szymanski, Anne-Marie Troost-Oppelaar, Faiz Z. Ramjankhan, Pim van der Harst, Monica Gianoli, Marish I. F. J. Oerlemans, Linda W. van Laake

**Affiliations:** 1 Department of Cardiology, University Medical Centre Utrecht, Utrecht, The Netherlands; 2 Transplantation Centre, University Medical Centre Utrecht, Utrecht, The Netherlands; 3 Department of Cardiothoracic Surgery, University Medical Centre Utrecht, Utrecht, The Netherlands

**Keywords:** Left ventricular assist device (LVAD), Heart failure (HF), Healthcare consumption

## Abstract

**Background:**

A left ventricular assist device (LVAD) is a life-saving but intensive therapy for patients with end-stage heart failure. We evaluated the healthcare consumption in a cohort of LVAD patients in our centre over 6 years.

**Methods:**

All patients with a primary LVAD implantation at the University Medical Centre Utrecht in Utrecht, the Netherlands from 2016 through 2021 were included in this analysis. Subsequent hospital stay, outpatient clinic visits, emergency department visits and readmissions were recorded.

**Results:**

During the investigated period, 226 LVADs were implanted, ranging from 32 in 2016 to 45 in 2020. Most LVADs were implanted in patients aged 40–60 years, while they were supported by or sliding on inotropes (Interagency Registry for Mechanically Assisted Circulatory Support class 2 or 3). Around the time of LVAD implantation, the median total hospital stay was 41 days. As the size of the LVAD cohort increased over time, the total annual number of outpatient clinic visits also increased, from 124 in 2016 to 812 in 2021 (*p* = 0.003). The numbers of emergency department visits and readmissions significantly increased in the 6‑year period as well, with a total number of 553 emergency department visits and 614 readmissions. Over the years, the annual number of outpatient clinic visits decreased by 1 per patient-year follow-up, while the annual numbers of emergency department visits and readmissions per patient-year remained stable.

**Conclusion:**

The number of patients supported by an LVAD has grown steadily over the last years, requiring a more specialised healthcare in this particular population.

**Supplementary Information:**

The online version of this article (10.1007/s12471-024-01885-5) contains supplementary material, which is available to authorized users.

## What’s new?

- The number of patients supported by a left ventricular assist device (LVAD) has grown steadily over the last years, requiring more specialised health care.
- In the Dutch LVAD cohort, we show for the first time that the numbers of outpatient clinic visits, emergency department visits and hospital readmissions significantly increased during the study period (2016–2021).
- Over the years, the annual number of outpatient clinic visits per patient-year follow-up decreased by 1, while the annual numbers of emergency department visits and readmissions per patient-year remained stable.
- Patients who received an LVAD at lower Interagency Registry for Mechanically Assisted Circulatory Support (INTERMACS) classification (especially when implanted while on temporary mechanical circulatory support) stayed significantly longer in hospital after implantation than those implanted at higher INTERMACS classification, highlighting the need for early referral for advanced heart failure therapy.
- In the future, general cardiologists will increasingly encounter patients supported by an LVAD.

## Introduction

When heart failure (HF) becomes progressive despite optimal guideline-directed medical therapy, advanced treatment options are available for carefully selected patients [1]. The first left ventricular assist device (LVAD) for the treatment of end-stage HF in the Netherlands was implanted at the University Medical Centre Utrecht (UMC Utrecht) in 1993 [2]. Due to the development and improved performance of continuous-flow devices, implantation numbers have risen over the last decade, with 4 Dutch centres now implanting LVADs. Current devices are implanted as bridge-to-transplant (~50% of the population), bridge-to-decision or destination therapy [3]. Due to the considerable shortage of donor organs in the Netherlands, LVAD implantation has become an important treatment strategy for patients with advanced HF [4]. Patients receiving LVAD therapy rely on networks of care that manage and treat them across multiple phases of the disease process. At the UMC Utrecht, a clinical pathway consisting of a dedicated multidisciplinary LVAD team is in practice.

In the current observational study, we evaluated the healthcare consumption of LVAD patients at our centre, focusing on a cohort of patients in the era of modern LVAD device types.

## Methods

As this study does not fall under the scope of the Dutch Medical Research Involving Human Subjects Act (*Wet medisch-wetenschappelijk onderzoek met mensen*), it does not require approval from an accredited medical ethics committee in the Netherlands. However, an independent quality check was carried out at the UMC Utrecht to ensure compliance with legislation and regulations (file number 20-058). In addition, at our hospital, patients with an LVAD registered informed consent at the UNRAVEL Research Data Platform were reviewed and accepted by the Medical Ethics Committee of the UMC Utrecht (number 12-387) [5].

### Inclusion criteria and endpoints

We retrospectively analysed a cohort of patients consisting of all-comers who underwent a primary LVAD implantation at the UMC Utrecht from 1 January 2016 through 31 December 2021. Patients receiving either the HeartMate 3 or HeartWare Ventricular Assist Device were included. Baseline characteristics and outcome data were collected using electronic health records.

Outcomes were hospital length of stay (LOS) before and after LVAD implantation, outpatient clinic visits, emergency department visits and readmissions at the UMC Utrecht. Patients were censored when they underwent heart transplantation during follow-up. The healthcare costs of this clinical pathway are outside the scope of this study.

### Statistical analysis

Baseline characteristics are presented as mean ± standard deviation (SD) or median with 95% confidence interval (CI) for continuous variables unless stated otherwise, and categorical variables are presented as number (%). Trends were assessed by testing potential associations for continuous outcomes over time (per year) using univariable linear regression. For associations based on categorical outcomes, the Fisher’s exact test was used. Differences for continuous variables between 2 groups were assessed by evaluating normality using QQ plots and then using the two-sample *t*-test (in case of normality) or Mann-Whitney U test (in case of non-normality). Total follow-up time in patient-years was calculated starting from the LVAD implantation until heart transplantation, death or the end of follow-up (31 December 2021). Kaplan-Meier curves were used to visualise survival after LVAD implantation, with patients being censored in the event of heart transplantation. A *p*-value < 0.05 was considered statistically significant. Statistical analyses were performed using SPSS Statistics Version 27.0.

## Results

### Healthcare consumption

**Fig. 1:**
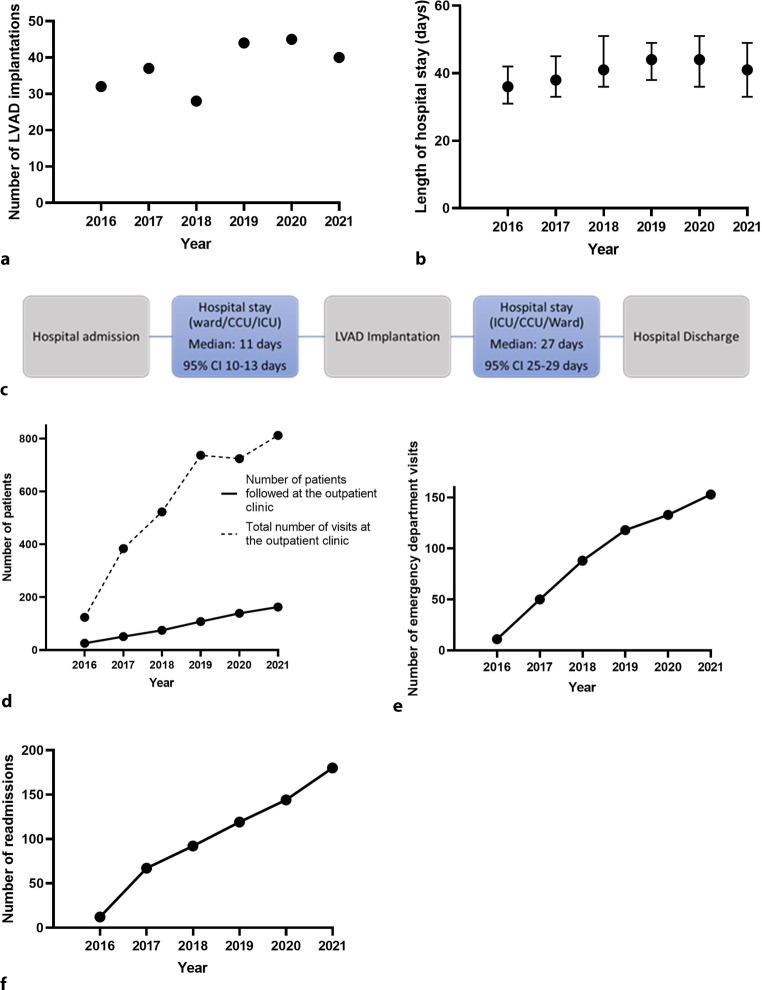
Healthcare consumption in patients undergoing left ventricular assist device (*LVAD*) in period 2016–2021. **a** Number of primary LVAD implantations. **b** Median length of hospital stay (pre- and post-LVAD implantation hospital stays combined). **c** Overview of LVAD implantation process. **d** Number of LVAD patients in outpatient clinic (bold line: β: 28; *p* < 0.001) and total number of outpatient clinic visits (*dashed line*: β: 114; *p* = 0.003). **e** Number of emergency department visits (β: 24; *p* = 0.003). **f** Number of readmissions (β: 31; *p* = 0.001)

From 2016 through 2021, a total of 226 primary LVAD implantations took place, either as bridge-to-transplant, bridge-to-decision or destination therapy. The annual number of implantations increased from 32 in 2016 to 45 in 2020 (*p* = 0.068) (Fig. 1a). The total LOS around implantation (including clinical screening/optimisation phase and post-operative course) was relatively constant over the years, with a median LOS of 41 days (95% CI: 37–43; *p* = 0.69) (Fig. 1b). Prior to LVAD implantation, the median LOS was 11 days (95% CI: 10–13), whereas the median LOS after LVAD implantation (intensive care unit and ward combined) was 27 days (95% CI: 25–29) (Fig. 1c). No differences were seen in pre- and post-LVAD implantation LOS over the years (data not shown).

Due to the increasing number of implantations in the cohort, the number of patients followed at the LVAD outpatient clinic increased steadily over the years. In 2016, a total of 26 individual patients who had received an LVAD since 1 January 2016 were followed compared with 163 patients in 2021 (β: +28 patients/year; *p* < 0.001) (Fig. 1d). This resulted in an increase in the number of outpatient clinical visits (defined as a visit to the cardiologist or specialised LVAD nurse) from 124 in 2016 to 812 in 2021 (β: +114 visits/year; *p* = 0.003) (Fig. 1d), ranging from 1 to 56 visits per patient, with a median of 13 visits during the study period. The annual number of outpatient clinic visits decreased by 1 per patient-year follow-up (B: −0.97; *p* = 0.002) (Tab. 1).

**Table 1:** Annual rates and rates per patient-year of emergency department visits, outpatient clinic visits and readmissions

Variable	2016	2017	2018	2019	2020	2021
Number of patient-years follow-up	12.8	40.2	62.4	90.8	123.1	150.1
Outpatient clinic visits	124	384	523	737	724	812
Outpatient clinic visits per patient-year follow-up^a^	9.7	9.5	8.4	8.1	5.9	5.4
Emergency department visits	11	50	88	118	133	153
Emergency department visits per patient-year follow-up	0.9	1.2	1.4	1.3	1.1	1.0
Admissions	12	67	92	119	144	180
Admissions per patient-year follow-up	0.9	1.7	1.5	1.3	1.2	1.2

Data are *n*; ^a^*P < 0.05*

The number of post-LVAD emergency department visits increased over time (β: +24 visits/year; *p* = 0.003) (Fig. 1e). In the 6‑year period, 161 individual LVAD patients visited the emergency department, resulting in a total of 553 emergency department visits (median: 2 visits/patient; range: 1–20). Nine patients visited the emergency department ≥ 10 times during the study period. Over time, no differences were found in the number of emergency department visits per patient-year follow up (B: 0.06; *p* = 0.96) (Tab. 1). Of the emergency department visits. 352 (64%) resulted in hospital admission. Patients presented primarily to the cardiologist or cardiothoracic surgeon (*n* = 429; 78% of presentations), followed by the neurologist (*n* = 40; 7%), gastroenterologist (*n* = 20; 4%) and the ear-nose-throat specialist (*n* = 20; 4%).

A total of 614 readmissions were recorded in the period 2016–2021. The annual number of readmissions of patients increased from 12 in 2016 to 180 in 2021 (β: 31; *p* = 0.001) (Fig. 1f). Over time, no differences were found in the number of admissions per patient-year follow up (B: −0.04; *p* = 0.94) (Tab. 1). A total of 154 individual patients with an LVAD were readmitted after implantation, ranging from 1 to 42 admissions per patient. The median time until the first emergency department visit was 147 days (95% CI: 122–209) after implant, whereas the median time until the first readmission was 167 days (95% CI: 117–209). Table S1 in the Electronic Supplementary Material illustrates the reasons for hospital admission.

### Patient population

**Fig. 2:**
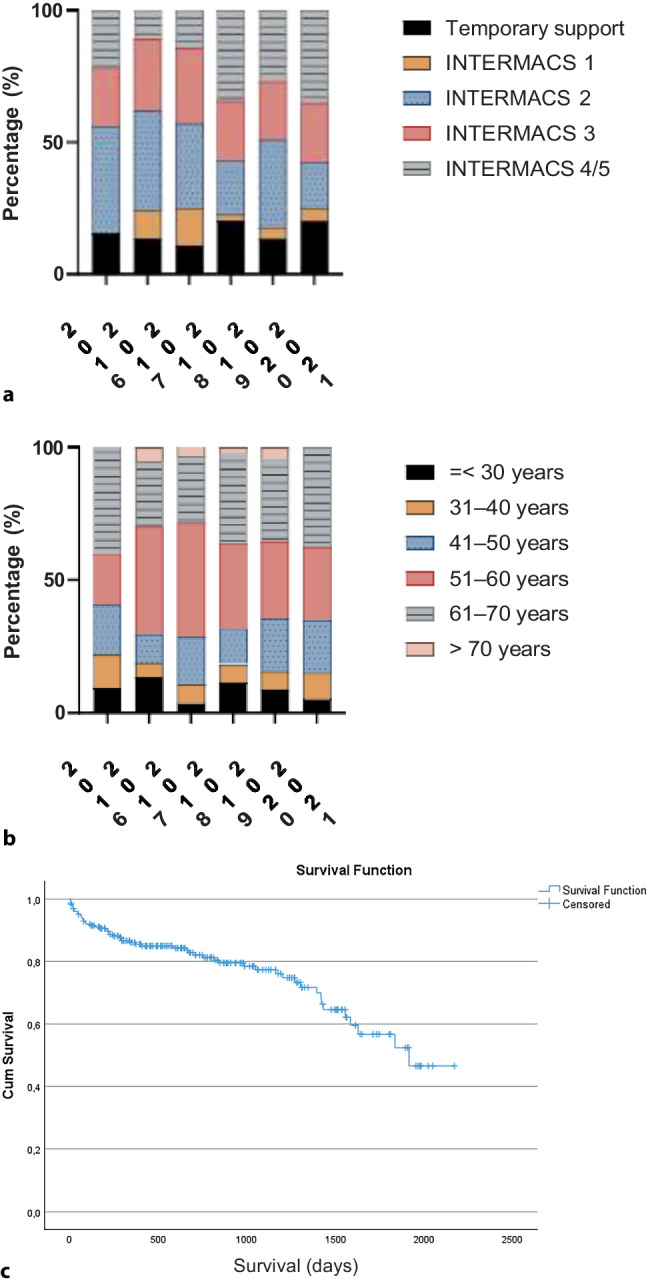
**a** Interagency Registry for Mechanically Assisted Circulatory Support (*INTERMACS*) classification at left ventricular assist device (*LVAD*) implantation. **b** Mean age at LVAD implantation. **c** Kaplan-Meier curve illustrating survival after LVAD implantation (patients censored after heart transplantation)

Patient characteristics are summarised in Tab. 2. Patients who received an LVAD in the period 2016–2021 were on average 53 ± 13 years of age; this mean age remained constant over the years (data not shown). Of the patients, 66% were male. The underlying aetiology of HF was ischaemic in 67 patients (30%). Most patients received an LVAD while being supported by inotropes. The Interagency Registry for Mechanically Assisted Circulatory Support (INTERMACS) class was 2 in 67 patients (30%) and 3 in 54 patients (24%). Temporary mechanical circulatory support was indicated in 36 patients (16%) in whom the LVAD was implanted. Over the years, no significant differences were seen in INTERMACS classification at LVAD implantation (*p* = 0.51) (Fig. 2a). There were no gender differences in INTERMACS class (*p* = 0.807): 12 female patients (16%) were implanted while on temporary mechanical circulatory support compared with 24 of the male patients (16%) (see Table S2 in Electronic Supplementary Material).

**Table 2:** Characteristics of patients with primary LVAD implantation in period 2016–2021

Variable	Patients (*N* = 266)
Age in years	53 ± 13
Male	149 (66)
*Heart failure aetiology*	
Ischaemic	67 (30)
Non-ischaemic	159 (70)
*INTERMACS class*	
T: temporary mechanical circulatory support	36 (16)
1: critical cardiogenic shock	13 (6)
2: progressive decline on inotropes	67 (30)
3: inotrope dependency	54 (24)
4/5: symptoms at rest or exertion intolerance	56 (24)

Data are mean ± standard deviation or *n* (%)

*LVAD* left ventricular assist device, *INTERMACS* Interagency Registry for Mechanically Assisted Circulatory Support

LVADs were mainly implanted in patients aged 51–60 years (*n* = 71; 31%) and those aged 61–70 years (*n* = 73; 32%). The youngest patient was implanted at age 16 years and the eldest at 74 years of age. The age distribution at implantation remained stable over the years (Fig. 2b), and the mean ± SD age did not differ between men (52 ± 14 years) and women (54 ± 12 years; *p* = 0.10). There was no significant difference in INTERMACS classification across different age categories (*p* = 0.31; see Table S3 in Electronic Supplementary Material). Of the patients aged ≤ 50 years, 30% were implanted while on temporary mechanical circulatory support or they had INTERMACS class 1, compared with 17% of those aged > 50 years.

The patients included in our cohort were referred to the UMC Utrecht for advanced HF care from all over the Netherlands. The 6 main referring regions were the provinces North Holland (*n* = 57; 25%), Utrecht (*n* = 45; 20%), Gelderland (*n* = 42; 19%), North Brabant (*n* = 32; 14%), Overijssel (*n* = 19; 8%) and South Holland (*n* = 13; 6%).

Figure 2c displays a Kaplan-Meier curve illustrating survival after LVAD implantation, with patients being censored in the event of heart transplantation.

### Differences in healthcare consumption

**Fig. 3:**
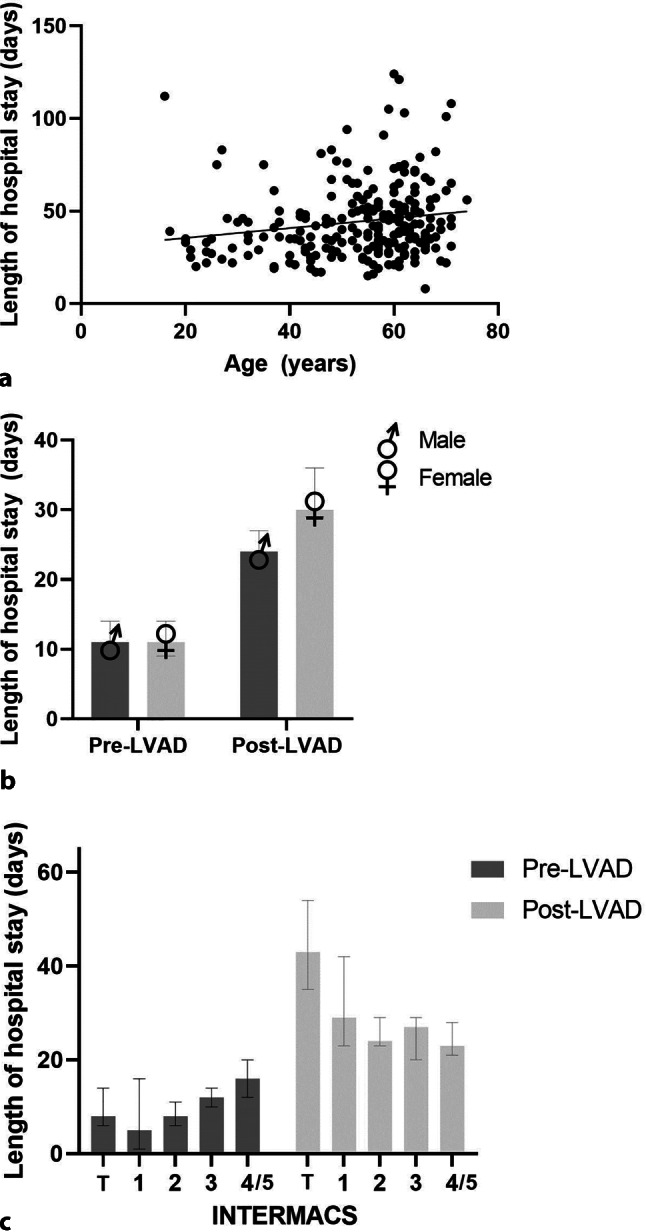
**a** Scatterplot of length of hospital stay around left ventricular assist device (*LVAD*) implantation (pre- and post-implantation) in relation to age (β: 0.26; R^2^: 0.03; *p* = 0.01). **b** Median length of hospital stay (with 95% confidence interval (CI)) pre- and post-LVAD implantation in men and women. **c** Median length of hospital stay (with 95% CI) in pre-LVAD implantation phase (*p* = 0.001) and post-LVAD implantation phase (*p* = 0.001) in relation to Interagency Registry for Mechanically Assisted Circulatory Support (*INTERMACS*) class. *T* temporary mechanical circulatory support

The median duration of hospital admission around the LVAD implantation was 41 days. Hospital stay was slightly longer with increasing age (β: +0.26 days/year; *p* = 0.01) (Fig. 3a). A similar trend was seen for duration of pre-LVAD and post-LVAD hospital stay (data not shown). A trend towards a longer hospital stay was observed for women compared with men, although this difference was not statistically significant (median: 46 days; 95% CI: 42–50 for women vs 38 days; 95% CI: 36–42 for men; *p* = 0.17). This was mainly determined by a longer post-LVAD implantation LOS in women than men (30 days; 95% CI: 29–36 vs 24 days; 95% CI: 23–27; *p* = 0.053). The pre-LVAD implantation duration was similar for men and women (11 days; 95% CI: 10–14 vs 11 days; 95% CI: 9–14; *p* = 0.68) (Fig. 3b).

The relation between the INTERMACS classification and duration of hospital admission is summarised in Fig. 3c and Table S4 in the Electronic Supplementary Material. Patients who received an LVAD while on temporary mechanical circulatory support (INTERMACS class T) stayed significantly longer in hospital (median: 60 days; 95% CI: 48–65) than patients with INTERMACS class 1, 2, 3 or 4/5 (median: 36, 36, 39 and 44 days, respectively; all *p* < 0.05)*.* Pre-LVAD LOS differed by INTERMACS class (*p* < 0.001). The median pre-LVAD hospital stay was 5 days (95% CI: 1–16) in patients with INTERMACS class and 16 days (95% CI: 12–20) in those with INTERMACS class 4 (*p* = 0.15). There was an inverse relation between post-LVAD implantation hospital stay and INTERMACS class (*p* = 0.001), which mean that the lower the INTERMACS class, the longer the post-LVAD hospital stay. Patients on mechanical supported stayed 43 days (95% CI: 35–54) in hospital compared with 23 days for those with INTERMACS class 4 (95% CI: 21–28; *p* = 0.001).

## Discussion

Since the start of the LVAD programme at the UMC Utrecht, the LVAD patient population has increased in size. This has resulted in more patients being monitored at the multidisciplinary outpatient clinic, more emergency department visits and more readmissions. On-device survival is estimated to be 83% at 1 year and 54% at 5 years [3, 6]. With half of the patients receiving an LVAD as bridge-to-transplant and an average of 15 heart transplantations at the UMC Utrecht per year during the analysed period (2016–2021), of which on average ~50% are on LVAD therapy when receiving the donor heart, the net LVAD patient population has increased over time and will likely continue to grow. This growing cohort of patients on LVAD therapy impacts our healthcare system. Therefore, improvements in this clinical pathway are constantly being implemented. For example, to reduce the duration of hospital stay after LVAD implantation, a collaboration with a rehabilitation clinic (Domstate in Utrecht, the Netherlands) was recently initiated, allowing earlier hospital discharge.

Over the years, there has been a decrease in the number of outpatient clinic visits by approximately 1 visit per patient-year, aligning with a reduction in scheduled visits due to increased experience. Importantly, this did not lead to changes in the number of emergency department visits or of readmissions. Post-LVAD readmission rates are high, ranging from 26% to 76% per year [7–9]. In our clinic, 68% of the patients were readmitted after LVAD implantation in the analysed time period. To reduce hospital readmissions, e‑health solutions such as monitoring and telemonitoring are currently being evaluated and implemented [10]. LVAD care needs a multidisciplinary approach aimed at reducing, among others, readmission rates and optimising adherence to self-care and the use of guideline-directed medical therapy [11, 12]. Therefore, our LVAD team consists of HF cardiologists and surgeons, specialised LVAD nurses, nurse practitioners, physician assistants, physiotherapists specialised in cardiac rehabilitation, medical social workers and dieticians [13]. Previous reports have shown that optimal management of LVAD patients benefits from integrated and coordinated care delivery. A well-organised structure and organisation of care is known to improve patient outcomes such as the infection rate [14]. More and more patients on LVAD therapy are treated in a single centre, which reduces total hospital costs per patient, possibly partially explained by better logistics and more experience [15].

### Age

The majority of LVAD patients received their LVAD at the age of 40–60 years. A smaller part of LVADs are implanted in a relatively older group of patients > 65 years. Data from other centres confirm that older patients (with possibly multiple co-morbidities) are increasingly accepted for LVAD implantation. In our population, hospital stay was only slightly longer with increasing age. A recent study showed comparable 30-day readmission rates in older and younger patients [16]. Therefore, general cardiologists should consider early referral of patients, including those of advanced age, for evaluation of LVAD therapy. However, it must be noted that this population represents a highly selected group of elderly patients. In our clinical pathway, all patients > 40 years of age are screened by a geriatrician, to assess the patient’s frailty and improve patient selection for advanced LVAD therapy [17, 18].

### Gender

Compared to other studies, our cohort contained a relatively large proportion (34%) of females. Women are less likely to receive an LVAD than men with similar clinical characteristics [19, 20]. In the first studies with pulsatile-flow LVADs, women were underrepresented. This was partly because of anatomical factors with devices being larger compared with the current continuous-flow LVADs. Since the introduction of continuous-flow LVADs, the number of women undergoing LVAD implantation has increased, but a gender gap still exists [21].

We did not observe any differences in age nor INTERMACS classification at implantation between men and women. While our study did not focus on patient outcomes, previous research showed that in-hospital outcomes in women are comparable to those in men, [20] although females showed increased mortality in the first months after LVAD implantation, partially driven by worsening right ventricular dysfunction and left ventricle–LVAD size mismatch [21].

### INTERMACS

Patients who received an LVAD at lower INTERMACS classification (especially when implanted while on temporary mechanical circulatory support) stayed significantly longer in hospital after implantation than those implanted at higher INTERMACS classification. This is consistent with previous reports demonstrating that the severity of cardiac failure preceding device implantation is one of the most important variables affecting LOS [22, 23]. This highlights the need for early referral of these patients for consideration of advanced HF therapy [24, 25]. In our cohort, no difference in INTERMACS classification at implantation was observed over the years.

## Conclusion

The number of HF patients supported by an LVAD steadily increased at the UMC Utrecht from 2016 through 2021, leading to increased healthcare consumption of this care-intensive patient group. As general cardiologists will increasingly encounter these patients, clinical pathways for the treatment of this specific population should be developed and introduced [24, 26].

### Supplementary Information

The supplemental tables contain an overview on reasons for hospital admission (supplemental table 1), the relation between gender and INTERMACS classification (supplemental table 2), the relation between age and INTERMACS classification (supplemental table 3) and the relation between INTERMACS classification and length of hospital stay (supplemental table 4)
