# Supplementary material for: Healthcare consumption of patients with left ventricular assist device: real-world data
Source: Neth Heart J. 2024 Aug 14;32(9):317–25. doi: 10.1007/s12471-024-01885-5 (PMC11336021; doi:10.1007/s12471-024-01885-5)
Supplement: Supplementary file 1 — The supplemental tables contain an overview on reasons for hospital admission (supplemental table 1), the relation between gender and INTERMACS classification (supplemental table 2), the relation between age and INTERMACS classification (supplemental table 3) and the relation between INTERMACS classification and length of hospital stay (supplemental table 4) [file 12471_2024_1885_MOESM1_ESM.docx]

| **Reason of admission** | **Number (percentage)** |
| --- | --- |
| Bleeding/ Thrombosis | 103 (21%) |
| Rhythm disorders/pacemaker or ICD implantation | 73 (15%) |
| Fever/infection | 70 (14%) |
| Not-LVAD related | 44 (9%) |
| LVAD surgery* | 37 (8%) |
| Cardiac decompensation | 34 (7%) |
| LVAD alarms | 31 (6%) |
| Gastro- or coloscopy | 27 (6%) |
| Other | 67 (14%) |
| * outflowgraft revisions, exit site revision, controler switch, LVAD switch | |

**Table S1**. Reasons for hospital admission (total n and percentage)

| **INTERMACS** | T |  | 1 |  | 2 |  | 3 |  | 4 |  | 5 |  |
| --- | --- | --- | --- | --- | --- | --- | --- | --- | --- | --- | --- | --- |
| **Gender** | n | % | n | % | n | % | n | % | n | % | n | % |
| Female | 12 | 16% | 6 | 8% | 19 | 25% | 25 | 33% | 14 | 18% | 1 | 1% |
| Male | 24 | 16% | 7 | 5% | 48 | 32% | 29 | 20% | 39 | 26% | 2 | 1% |

**Table S2.** Gender and INTERMACS classification at implantation (total n and percentage)

| **Age** | =< 30 years | | 31-40 years | | 41-50 years |  | 51-60 years |  | 61-70 years |  | > 70 years |  |
| --- | --- | --- | --- | --- | --- | --- | --- | --- | --- | --- | --- | --- |
| **INTERMACS** | n | % | n | % | n | % | n | % | n | % | n | % |
| T | 4 | 11% | 3 | 8% | 8 | 22% | 10 | 28% | 11 | 31% | 0 | 0% |
| 1 | 3 | 23% | 2 | 15% | 3 | 23% | 4 | 31% | 1 | 8% | 0 | 0% |
| 2 | 5 | 8% | 6 | 9% | 13 | 19% | 22 | 33% | 19 | 28% | 2 | 3% |
| 3 | 5 | 9% | 2 | 4% | 5 | 9% | 18 | 33% | 22 | 41% | 2 | 4% |
| 4 | 3 | 5% | 5 | 9% | 9 | 16% | 17 | 30% | 20 | 36% | 2 | 4% |

**Table S3.** Age and INTERMACS classification at implantation (total n and percentage)

|  | Total length of stay | | | | Pre-LVAD length of stay | | | | Post-LVAD length of stay | | | |
| --- | --- | --- | --- | --- | --- | --- | --- | --- | --- | --- | --- | --- |
| **INTERMACS** | Median | 95,0% Lower CL for Median | 95,0% Upper CL for Median | n | Median | 95,0% Lower CL for Median | 95,0% Upper CL for Median | n | Median | 95,0% Lower CL for Median | 95,0% Upper CL for Median | n |
| T | 60 | 48 | 65 | 36 | 8 | 6 | 14 | 36 | 43 | 35 | 54 | 36 |
| 1 | 36 | 29 | 46 | 13 | 5 | 1 | 16 | 13 | 29 | 23 | 42 | 13 |
| 2 | 36 | 33 | 41 | 67 | 8 | 6 | 11 | 67 | 24 | 23 | 29 | 67 |
| 3 | 39 | 35 | 44 | 54 | 12 | 10 | 14 | 54 | 27 | 20 | 29 | 54 |
| 4 | 44 | 35 | 47 | 56 | 16 | 12 | 20 | 56 | 23 | 21 | 28 | 56 |

**Table S4**. INTERMACS classification in relation to length of hospital stay (LOS) (total, pre- and post LVAD) (median ± 95% CI)
